# A Monte Carlo simulation based on first-passage distributions for spatio-temporal detection on potentiometric sensor arrays

**DOI:** 10.1007/s00216-026-06643-7

**Published:** 2026-07-14

**Authors:** Lewis Keeble, Kunal Katarya, Ahmad Moniri, Nicolas Moser, Pantelis Georgiou, Jesus Rodriguez-Manzano

**Affiliations:** 1 Department of Electrical and Electronic Engineering, Imperial College London, London, SW7 2AZ UK; 2 Department of Infectious Disease, Imperial College London, London, W12 0NN UK

**Keywords:** ISFET, Potentiometric, Sensor array, Monte Carlo simulation, Electrochemical detection, Spatio-temporal

## Abstract

**Abstract:**

This paper presents a novel simulation approach for particle diffusion that uses Monte Carlo sampling of first-passage distributions to conduct spatio-temporal modelling for potentiometric sensing arrays. The Monte Carlo first-passage (MCFP) simulation algorithm prioritises speed to overcome limitations with currently available techniques. This paper applies the MCFP technique to simulate the detection of protons by arrays of ion-sensitive field-effect transistors (ISFETs) as a case study. The MCFP algorithms were validated against a benchmark random walk algorithm, with temporal MCFP output matching that of the random walk with an average $r^2$ value of 0.901. The simulations were further validated against experiments using a microchip-based ISFET array to observe a localised pH change created by an acid-containing glass capillary held at various heights from the sensing surface. MCFP simulations displayed an ability to accurately estimate the mean array output over time with an average $r^2$ value of 0.749±0.170 and a maximum of 0.986, whilst increasing simulation speed by over at least an order of magnitude compared to that of the random walk. MCFP simulations recreated several key trends of spatial signal patterns, with the proportional increase in the variance of the Gaussian consistent to within two standard deviations between experiments and simulations in all eight cases. MCFP simulations underestimated the variance in seven out of eight cases, likely due to fluid motion within the experimental setup, but were consistent to two standard deviations with experimental values for all four vertical array cross-sections.

**Graphical abstract:**

**Figure:**
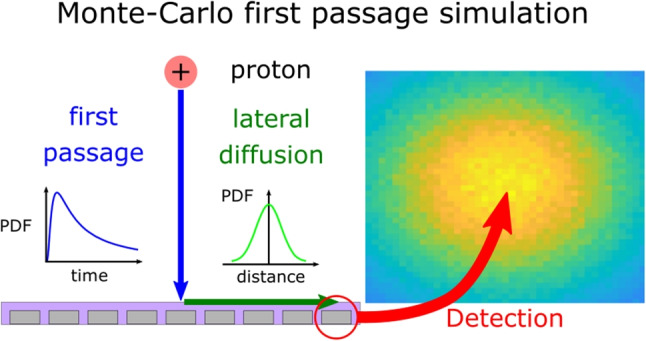

This research article presents a novel Monte Carlo-based simulation approach for the detection of electrochemical species by potentiometric sensor arrays that combines first-passage time sampling with diffusion to capture spatio-temporal signals. This approach is validated using a CMOS-based ISFET array to detect pH changes from acid-filled glass capillaries

## Introduction

Potentiometric sensors, which measure the electric potential between a working and reference electrode with no current flowing, are a subset of electrochemical sensors that have become popular for a wide range of applications, including biomedical sensing, environmental monitoring, and food quality assessment [1]. Key to the rise in the usage of potentiometric sensors is their compatibility with complementary metal-oxide-semiconductor (CMOS) technology, providing low-power and scalable sensing solutions. This has led to the creation of low-cost, portable sensing platforms that circumvent logistical and resource limitations.

Since their first creation, the signal analysis of potentiometric sensing devices has been largely limited to the temporal output, classifying electrochemical detection events from changes in electric potential over time [2]. However, as the technology has advanced and prices scaled down, potentiometric sensors are now routinely integrated as part of large-scale sensing arrays on microchips that contain tens of thousands of pixels [3], whilst the frame rate for array readout is ever-increasing [4]. These two factors provide the opportunity to exploit a new dimension—space—and combine it to perform spatio-temporal signal analysis.

Models are required to develop our fundamental understanding of potentiometric sensing through simulation, which will help to identify spatio-temporal features for exploitation, inform the development of signal processing algorithms, and guide the design of sensors on the array level. However, currently available simulations of potentiometric sensing are insufficient for array-level spatio-temporal simulation. They typically involve workarounds in SPICE [5–8] or TCAD [9, 10] that replicate the electrolyte as a semiconductor element with chemically dependent characteristics, and are almost entirely focussed on the single-sensor level.

Modelling the spatio-temporal signals across a potentiometric sensor array necessitates simulating the diffusion of the electrochemical species of interest from their point of origin to the sensors. The most established simulation approach for particle diffusion is the random walk, where the diffusive movement of particles is tracked over small time steps [11, 12]. This process is computationally intensive due to the small time steps required to accurately track many fast-diffusing electrochemical species over long times. Finite-element method (FEM) simulations scale with the number of mesh elements, rather than particles [13]. However, in simulating a system that is large relative to the size of a single pixel, the runtime can still be prohibitively long.

This manuscript will present a new simulation approach for particle diffusion that prioritises speed, by randomly sampling the time it takes electrochemical species to reach the sensing surface from the first-passage distribution. The first-passage time can then subsequently be used to sample the electrochemical species’ lateral displacement, creating a spatial dependence of the electric potential. This Monte Carlo first-passage (MCFP) approach will sacrifice some physicality through approximations, but will be able to simulate large and complex electrochemical sensing systems quickly, whilst providing spatio-temporal output. Furthermore, the independence of each time step means that the simulation can be split across a computing cluster for faster execution. Existing Monte Carlo simulations for potentiometric sensing either focus on the electrolyte-electrode interface and the interaction of electrochemical species with surface sites [14] or treat the test sample as homogeneous, thus neglecting spatial variations of signal [15].

The ion-sensitive field-effect transistor (ISFET), which detects protons and thus measures changes in solution pH, will be used as a demonstrator for the MCFP simulation. After being validated against a benchmark random walk algorithm, MCFP simulations will be compared against experimental data from an ISFET array using an acid-filled glass capillary to create a localised pH change. These experiments are designed to create a system that would be straightforward to model and thus provide a reliable means of validating the MCFP simulation approach, rather than presenting the ideal use case. These experiments could aptly be described using an analytical approach or could be simulated within a FEM software.

ISFET arrays have become a powerful sensing platform for infectious disease diagnosis [16], through detection of pH changes caused by DNA amplification reactions [17], and were hence chosen as the case study for this work. The spatial signals stemming from DNA amplification reactions are not well understood, but are expected to be highly correlated to the position of target DNA when the amplification reaction is initiated [18]. Work has begun to utilise these spatial signals to increase the speed of DNA detection by isolating regions of high activity on ISFET arrays [19]. Further harnessing the spatial component of ISFET array output, aided by the simulations developed in this manuscript, will pave the way to absolute quantification of DNA within samples, enabling rapid and cost-effective prognosis at the point-of-care. This will help to reconfigure global healthcare systems to provide better health for all.

## Experimental section

### ISFET pH sensing

The ISFET is a chemically sensitive analogue of the metal-oxide-semiconductor field-effect transistor (MOSFET), where the gate electrode is replaced by an insulating membrane that is in contact with an electrolyte and the system is biased by a reference electrode. The insulating surface contains amphoteric *OH* groups that can transition between three states: deprotonated ($O^-$), neutral (*OH*), and protonated ($OH_2^+$). This thus describes the two-site binding model [20].

In response to the surface charge that arises at the insulator-electrolyte interface from these surface reactions, counterions within the electrolyte will move to form an electrical double layer (EDL) [21–23]. The EDL consists of two main layers:

- **Helmholtz plane** (inner and outer): a planar layer of counterions that is analogous to a parallel plate capacitor ($C_{Helm}$) with a linear decrease in potential
- **Diffuse layer**: a continuous layer of charge that incorporates diffusive particle motion and exhibits an exponential decay of potential over the Debye length

**Fig. 1:**
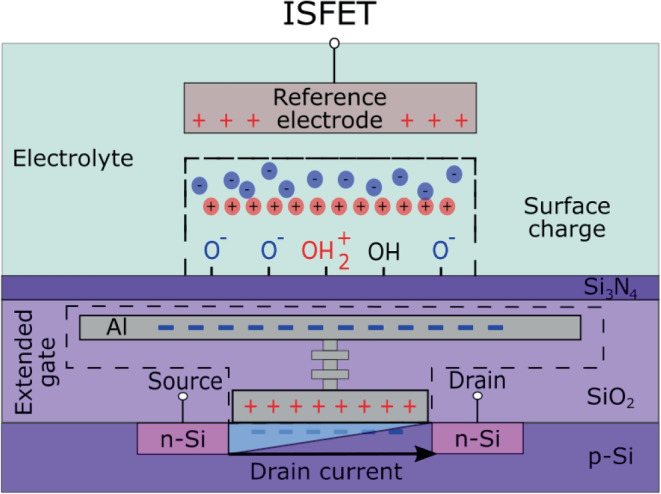
A diagram illustrating the various components of an ISFET and its sensing mechanism

The total potential difference across the EDL is given by

1 $$\begin{aligned} \psi _0 = \frac{2k_bT}{e}\text {sinh}^{-1}(\frac{-\sigma }{\sqrt{8c\epsilon _s\epsilon _0k_bT}}) + \frac{\sigma }{C_{Helm}} \end{aligned}$$

where σ is the surface charge density, $k_b$ is Boltzmann’s constant, *c* is the ionic concentration, and $\epsilon _s$ and $\epsilon _0$ are the relative medium and vacuum permittivity respectively (Fig. 1).

As the ionic content of the electrolyte changes, it modifies the surface charge and EDL at the insulator-electrolyte interface, which in turn modulates the drain current of the underlying MOSFET in a measurable fashion. The change in potential as measured by the ISFET can be described by the equation

2 $$\begin{aligned} V_{Chem} = \gamma + \alpha S_NpH \end{aligned}$$

where γ is a collection of constants that do not vary with ionic concentration and α is a constant, varying from 0 to 1, that dictates the deviation of the system from the Nernstian sensitivity, $S_N=59\,mV/pH$ at ambient temperature.

In practical usage, the ISFET suffers from several non-idealities. Those that are most relevant for this work are as follows:

- **Trapped charge:** A random threshold voltage offset caused by the trapping of charge between each layer deposited during the fabrication process [24].
- **Temporal drift:** A monotonic change in ISFET threshold voltage over time, caused by ionic bonding at buried *OH* sites within the insulating layer. The drift in threshold voltage, $\Delta V_{Th}$, follows an exponential function of time, *t*, described by
  3 $$\begin{aligned} \Delta V_{Th} = \Delta V_{Th}^\infty (1-exp(-\frac{t}{\tau })^\beta ) \end{aligned}$$
  where $\Delta V_{Th}^\infty$, τ, and β are parameters that are fit to experimental data [25].

### Proton diffusion

For a proton to be detected by an ISFET, it must diffuse from where it is produced down to the sensing surface. The probability that a diffusing particle occupies the position x+Δx at time t+Δt is the Gaussian distribution

4 $$\begin{aligned} P=\frac{1}{\sqrt{4\pi D\Delta t}}exp(-\frac{\Delta x^2}{4D\Delta t}) \end{aligned}$$

where *D* is the diffusivity of the particle [26]. The mean square displacement (MSD) measures the deviation of the particle over time and is linked to the variance of the Gaussian distribution ($\sigma ^2$) according to $MSD=2D\Delta t=\frac{1}{2}\sigma ^2$. Protons have a larger than expected diffusion coefficient of $1\times 10^{-8}\,m^2s^{-1}$ when moving through water owing to the Grotthuss mechanism [27].

In 1D, given a starting point, *x*, at time *t*, the probability that a particle reaches a point, x+Δx, at time, t+Δt, is given by the first-passage distribution, which is a Fourier series [28]. A simplified expression is obtained for a semi-infinite boundary condition

5 $$\begin{aligned} P_{FP}=\frac{\Delta x}{\sqrt{4\pi D}\Delta t^{3/2}}exp(-\frac{\Delta x^2}{4D\Delta t}) \end{aligned}$$

with the upper limit in *x* set to infinity.

Over a time step, Δt, the displacement of a particle due to diffusion is given by the Smoluchowski equation

6 $$\begin{aligned} \Delta x = \sqrt{2D\Delta t}N \end{aligned}$$

where *N* is a unit mean, unit variance ($\sigma ^2$) normally distributed random number and Δt must be significantly smaller than the characteristic mixing time [29]. The characteristic mixing time is

7 $$\begin{aligned} t_m=\frac{L^2}{4D} \end{aligned}$$

where *L* is the characteristic dimension of the chamber. A system evolving over a time longer than $t_m$ is considered to be well-mixed, and the concentration of particles is uniform. The mixing time for protons within a reaction chamber with dimension $L=2\times 10^{-3}\,m$ is roughly 100s.

The diffusive particle flux, *J* ($mol/m^2s^{-1}$), between two mixtures of particles at different concentrations, Δc, is described by Fick’s $1^{st}$ law of diffusion,

8 $$\begin{aligned} J=-D\Delta c \end{aligned}$$

### Simulation algorithms

#### MCFP

To randomly sample the first-passage distribution based on the *z*-coordinate of a proton’s origin, the cumulative density function (CDF) is obtained by integrating Eq. 5 and rearranging to form

9 $$\begin{aligned} \Delta t = (\frac{\Delta z}{erf^{-1}(-U_1)2\sqrt{D}})^2 \end{aligned}$$

where $U_1$ is a uniform random variable between 0 and 1 and $erf^{-1}$ is the inverse error function.

The *x* and *y* displacements can then be sampled using Δt, with particles only considered to reflect once to simplify the equations. This is valid since for secondary reflections to be prominent, the PDF of particle displacement will be flat, and thus, there will be little impact on the spatial output. By using Eq. 4 and superimposing an image PDF across each reflecting boundary, the resulting PDF is

10 $$\begin{aligned} P_{Ref}=exp(-\frac{(x-x_p)^2}{\sqrt{(4D\Delta t)}}) + exp(-\frac{(x+x_p)^2}{\sqrt{(4D\Delta t)}}) + exp(-\frac{(x+x_p-2l)^2}{\sqrt{(4D\Delta t)}}) \end{aligned}$$

where $x_p$ is the starting coordinate of the proton and the two boundaries are positioned at x=0 and x=l. When $\Delta t\ll t_m$, this equation reduces to Eq. 4.

The CDF, obtained by integrating Eq. 10, is

11 $$\begin{aligned} C(x,t)= &   \frac{1}{2}-\frac{1}{2}erf(-\frac{x-x_p}{\sqrt{4D\Delta t}})+\frac{1}{2}erf(-\frac{x+x_p}{\sqrt{4D\Delta t}})\nonumber \\  &   -\frac{1}{2}erf(-\frac{x+x_p-2l}{\sqrt{4D\Delta t}}) \end{aligned}$$

where *erf* is the error function. When $\Delta t\ll t_m$, this gives

12 $$\begin{aligned} x = erf^{-1}(2U_2-1)\sqrt{4D\Delta t} \end{aligned}$$

where $U_2$ is a uniform random variable between the limits $U_{LL}$ and $U_{UL}$, which are the values of $U_2$ when $x=-x_{p}$ and $x=l-x_{p}$, respectively.

Equation 11 must be solved numerically, which is slower than the analytical solution provided by Eq. 12. Identifying protons that are unlikely to interact with the boundaries within their first-passage time, and thus can have their displacement sampled using Eq. 12, will reduce simulation runtime. The maximum error between Eqs. 11 and 12 occurs at the boundary and as Δt tends to zero, thus defining the equation

13 $$\begin{aligned} A = -erf^{-1}(p_x-1) \end{aligned}$$

Any protons whose distance from the nearest boundary is greater than $A\sqrt{4D\Delta t}$ will have their displacement sampled using Eq. 12.

#### Random walk

A random walk simulation was developed to act as a benchmark against which the MCFP simulations could be compared. Tracking the random walk of a particle requires randomly sampling the Smoluchowski equation (Eq. 6) three times using unit mean, unit variance normal variables with $\Delta t\ll t_m$ and updating the *x*, *y*, and *z*-coordinates of the proton. If a proton’s updated *x* or *y* coordinates are outside the boundaries of the reaction chamber, the proton is moved to the intersection point of its diffusive trajectory with the wall. When a particle collides with the z=0 plane, its coordinates are saved, and the proton is removed from the simulation.

A key limitation of both MCFP and random walk simulation approaches is that they do not account for equilibrium phenomena, and given infinite timescales, all protons will be absorbed by the microchip surface, which is not physical. Within the pH range over which ISFETs typically operate, 2-10, and with monotonic pH decrease, the change in surface charge is dominated by transitions from $O^-$ to *OH*. Thus, the simulations will provide a consistent net description until proton production ceases and equilibrium is reached. Within this simplified representation, the number of protons absorbed at each sensor is linearly proportional to the change in surface charge density and thus, in accordance with Eq. 1 for small surface charges, is linearly proportional to the chemical potential at the surface of the microchip.

### Simulation methodology

Simulations were written and executed in MATLAB 2023b on a computer with an Intel Core i7 (4.20GHz) processor and 64Gb of RAM.

Benchmarking simulations were run on $1\times 10^5$ protons, using a 0.01s time step for the random walk simulations. The first set of simulations was conducted without boundary conditions, with proton release points at z=(0.25,1.25,2.25)mm and at x=1.1mm and y=0.75mm. Reflecting sidewalls were introduced in the second set of simulations at the x=(0,2.2)mm planes and the y=(0,1.5)mm planes with the same release points as the first set of simulations. The third set of simulations uses the same reflecting sidewalls with proton release points at z=1.25mm and x=(0.22,0.55,1.10)mm. A reflecting ceiling was added to the fourth set of random walk simulations at z=2.5mm. All benchmarking MCFP simulations were conducted with the CDF error tolerance set to 1%.

### Experimental methodology

The microchip used in experiments contains a 204×290 ISFET array, utilising the sensor design in Moser et al. 2018 [3], which performs time-domain encoding. The microchip has dimensions of $12\,mm\times 8\,mm$, with a pixel size of $35\,\mu m\times 35\,\mu m$ and separation of $2\,\mu m$.

**Fig. 2:**
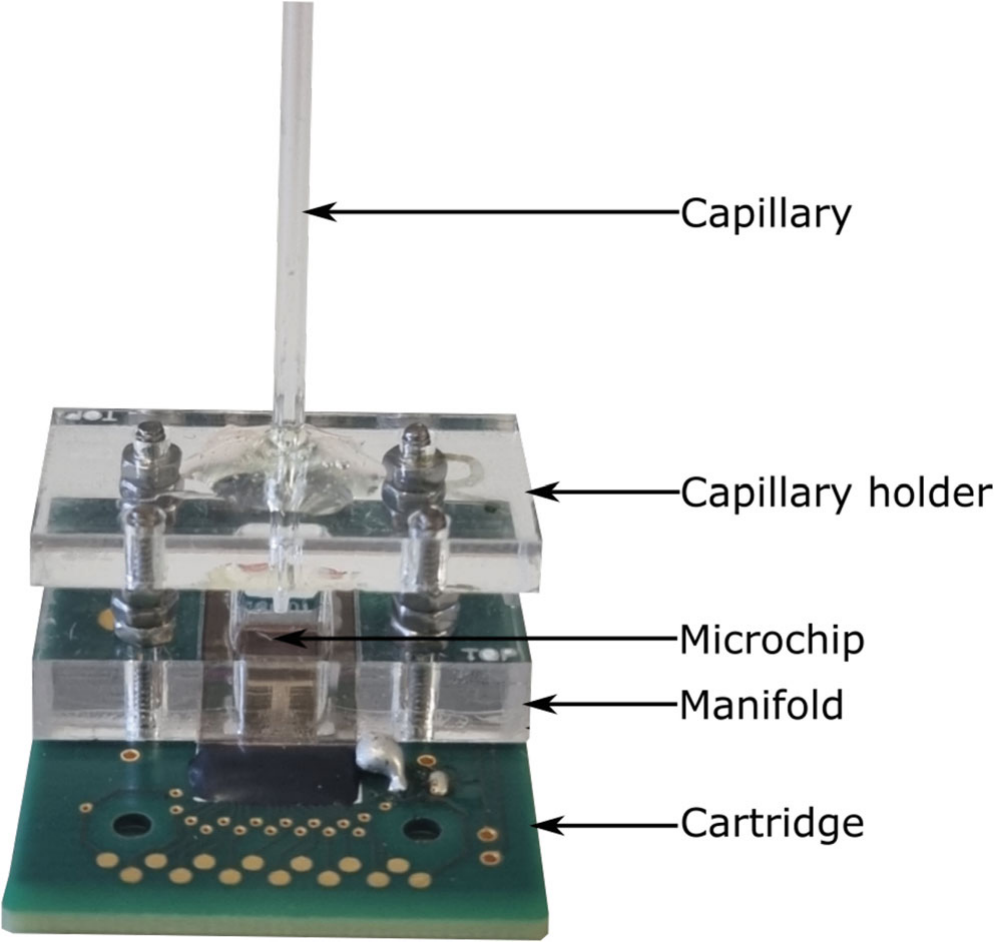
A photograph of the experimental setup used for experiments, showing the capillary, capillary holder, microchip, manifold, and cartridge

The microchip is glued to a printed circuit board (PCB) cartridge with conductive silver epoxy (Epotek), wirebonded (F&S), and encapsulated with glob-top epoxy (Epotek). The reaction well is defined by a laser-cut 5mm acrylic manifold with a $4.6\times 9.4\,mm$ opening and double-sided adhesive tape (Tesa) used to seal it. An Ag/AgCl quasi-reference electrode is produced by electrochemical oxidation of $25\,\mu m$ diameter silver wire (Goodfellows) in 1M KCl (Merck) by applying 1V in combination with a 1mm thick silver wire (Goodfellows) cathode. This reference electrode is incorporated within the manifold, between the adhesive tape and the microchip surface (Fig. 2).

The assembled microchip cartridge connects to a motherboard for microchip communication and data readout. The microchip is checked for correct functionality, active pixels are identified using a reference voltage step, these pixels are calibrated, and then the array readout begins.

To produce a highly localised change in pH, a glass capillary was used with an inner diameter of $680\,\mu m$. The capillary was glued to a laser-cut acrylic holder using quick-set epoxy (RS) with the height ‘zeroed’ to the microchip surface. Then, washers were used as spacers to raise the height of the capillary to 0.6mm and 1.2mm from the microchip surface.

**Table 1:** $r^2$ values comparing cumulative protons absorbed against time for MCFP and random walk simulations

	$$\textrm{r}^{2}$$		
z-coordinate	Without boundaries	Reflecting boundaries	
(*mm*)		No ceiling	With ceiling (normalised)
0.25	0.9991	0.9974	0.9692
1.25	1.0000	0.9989	0.9387
2.25	0.9999	0.9995	0.7964

The microchip well and glass capillary were filled with 100mM KCl and 100mM HCl, respectively, with solution pH measured with a calibrated pH probe (Sentron). The manifold was filled to the top of the well, the capillary was filled under capillary action, and the extent of the liquid rise was measured. This ensured that there was no liquid motion under capillary action when the capillary was added to the test solution. All experiments were conducted at an ambient temperature of 21 $^{\circ }$C.

At the beginning of each experiment, a 300s settling period was observed to minimise drift and provide sufficient datapoints to perform drift correction. After this point, the capillary was added to the system.

To simulate this system using the MCFP algorithm, the number of protons diffusing from the tip of the capillary at each time step was calculated using Eq. 8, and the pH of the two solutions was continuously updated. A CDF error tolerance of 10% was used.

### Data processing

The output from the microchip was linearised to translate the time-encoded values, $V_{time}$, into ISFET chemical potential, using an equation of the form $V_{chem}=D-\frac{1}{B}{}log(\frac{V_{time}-C}{A})$, where constants *A*, *B*, *C*, and *D* have been experimentally determined for the microchip being used through fitting of output with a sweep in reference potential.

Each pixel’s output was subtracted by its initial reading to account for trapped charge offset. The mean output across the array over time was compensated for drift by fitting Eq. 3 to the first 5 min of output, before the capillary was inserted. This fit was then subtracted from the output for all subsequent time points. All data was normalised against the maximum value for the mean signal across the array. The simulated data was fit to experimental data in the time dimension by finding the time offset that maximised the $r^2$ value between the two curves.

The drift-compensated mean signal from the experiments was differentiated, the peak identified ($t_1^{exp}$), and the half-width at half maximum (HWHM) determined, with $t_2^{exp}=t_1^{exp}+HWHM$. The start point of the signal, $t_{start}$, is taken as the first point that is above 2% of the peak value. The time points sampled from the simulated data for comparison are taken as $t_i^{sim}=t_i^{exp}-t_{start}$.

The spatial peaks within the 2D sensor array were identified at both $t_1$ and $t_2$ for experimental and simulated data, either using a peak finding function or user input. Then, for both horizontal and vertical cross-sections centred on the peak, Gaussian fits were made to the sensor output against pixel number, within a specified window to omit outliers near the periphery of the array.

The different drift rates of each sensor add noise to the spatial signals on the array. Individual sensor drift compensation will markedly improve the clarity of spatial patterns; however, this has proved challenging to perform due to the amount of noise on pixel-level signals and is further compounded by the pH-dependent characteristics of drift [30].

The different temporal drift rates of each sensor contribute to a corresponding positive, decaying exponential drift in standard deviation from zero. This was compensated for using an equation of the form $y=A(1-exp(-\frac{x}{B})^C)$.

## Results and discussion

### Benchmarking

#### No boundary conditions

The simulated output without any boundary conditions shows excellent agreement between the MCFP and random walk models, achieving an $r^2$ value greater than 0.999 for cumulative proton absorption over time—equivalent to the cumulative density function of first-passage times—at all three heights of proton release, as seen in Table 1. The $\sigma ^2$ of the Gaussian fits to the histograms of spatial distribution of proton absorption, collated in Table 2, are also consistent to two significant figures. This demonstrates that the MCFP algorithm has been correctly implemented and is producing physical results.

**Table 2:** $\sigma ^2$ of the normal distributions fitted to the *x*-coordinate of absorbed protons for random walk and MCFP simulations. Values stated are in units of $1\times 10^{-4}$

	$${\sigma ^2}$$				
	Without boundaries		Reflecting boundaries		
z-coordinate	MCFP	Random walk	MCFP	Random walk	
(*mm*)				No ceiling	With ceiling
0.25	11.961	11.951	3.236	3.279	3.321
1.25	20.636	20.632	6.671	6.570	7.095
2.25	29.748	30.059	53.153	119.301	92.518

**Table 3:** The mean (μ) and variance ($\sigma ^2$) of the Gaussians fit to the *x*-coordinate of absorbed protons in the third set of simulations as described in ‘Simulation methodology’

*x*-coordinate	MCFP		Random walk	
(*mm*)	μ	σ	μ	σ
		($\times 10^{-4}$)		($\times 10^{-4}$)
0.22	$1.283\times 10^{-7}$	12.400	$2.339\times 10^{-14}$	11.248
0.55	$1.688\times 10^{-4}$	12.823	$2.164\times 10^{-4}$	11.431
1.10	$1.140\times 10^{-3}$	6.671	$1.097\times 10^{-3}$	6.570

#### Reflecting sidewalls (without ceiling)

The temporal signals between the MCFP and random walk simulations showed similarly close agreement when sidewalls were introduced, with $r^2$ values greater than 0.9975 for all proton release points, as shown in Table 1. This is to be expected as the sidewalls do not impact the first-passage times of the protons.

The values for $\sigma ^2$ of the Gaussian fits with reflecting sidewalls are provided in Table 2. The spatial distributions simulated with the MCFP approach differ from those of the random walk simulation, with a difference in $\sigma ^2$ of under 2% for *z*-coordinates of 0.25mm and 1.25mm. The values of $\sigma ^2$ for the proton release point of 2.25mm are unreliable because of the low number of protons hitting the z=0 plane and the flat distribution leading to poor Gaussian fitting. Importantly, the MCFP simulation is able to recreate the spatial distributions from random walk simulations with acceptable accuracy for proton start positions that are close to the boundaries. As shown by the $\sigma ^2$ values contained in Table 3, the error between MCFP and random walk simulations increases to up to 12.2% as the proton release points move towards the sidewall.

These results demonstrate that the boundary conditions have been correctly implemented and the approximations that have been made have an acceptably small effect on the output.

#### Reflecting sidewalls (with ceiling)

**Fig. 3:**
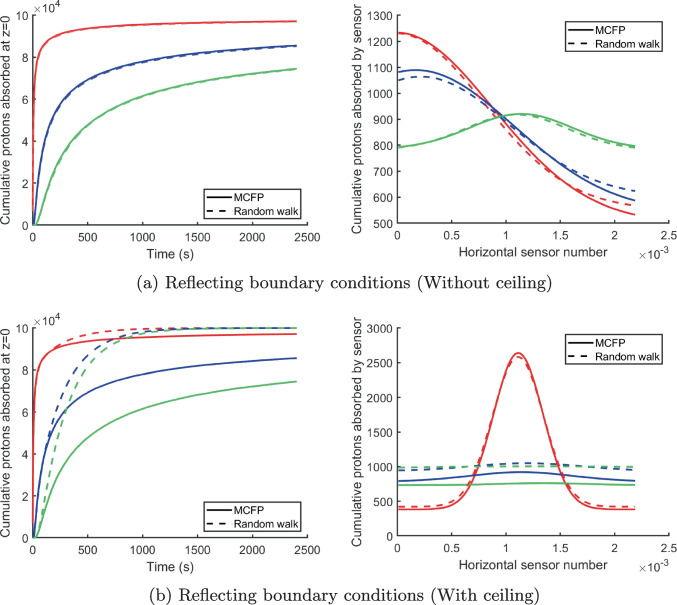
A comparison of temporal (left) and spatial (right) results for protons absorbed at the z=0 plane for the random walk (dashed lines) and MCFP (solid lines) simulations. **a**) the third set of simulations, as defined in ‘Simulation methodology’, with starting *x*-coordinates of (red) x=0.22mm, (blue) x=0.55mm, and (green) x=1.10mm. **b**) The fourth set of simulations, with starting *z*-coordinates of (red) z=0.25mm, (blue) z=1.25mm, and (green) z=2.25mm. The figures on the right contain normal distributions fitted to histograms of the *x*-coordinate of protons absorbed at the z=0 plane with 100 bins

The results displayed in Fig. 3b indicate that the closer protons are generated to the ceiling, the larger the discrepancy between the random walk and MCFP simulations. With a release point closer to the sensing plane, fewer protons are expected to interact with the upper boundary, and thus, the underlying differences in the models due to the semi-infinite approximation are less evident. The MCFP simulation performs well up until 100s, which is the mixing time of the system, at which point the difference in absolute output varies between 0.59-4.12%. The error in the MCFP simulations increases as time progresses, due to the protons being more likely to interact with the ceiling. The MCFP output at 2400s varies between 3 and 35% lower than the random walk simulation. When normalising the curves against their maximum value, the MCFP and random walk simulations have $r^2$ values over 0.796 for all three heights, which rises to over 0.939 for half the ceiling height and below, as seen in Table 1.

The discrepancy in first-passage times created by the addition of a ceiling has a knock-on effect on the spatial signals, as seen in Table 2, with $\sigma ^2$ increasing for random walk simulations and reducing their agreement with MCFP simulations, with up to a 6.4% difference for the simulations at z=2.25mm.

These results demonstrate that despite the large errors in the absolute output introduced by the semi-infinite approximation, particularly beyond the mixing time of the system, the temporal signals are replicated sufficiently well by MCFP simulations in terms of shape. As such, normalised output will be used for all subsequent tests.

The limitations of the MCFP simulation approach will be most apparent for systems evolving beyond their characteristic mixing time, which favours systems that are large and particles with low diffusivity relative to the simulated timespan. Larger errors will also be accrued for protons originating from nearer to the ceiling. However, for spatio-temporal signal detection, it is the protons closer to the sensing plane that are of more interest, as these contribute more to the early signal and form stronger spatial patterns due to reduced lateral diffusion, which means that the MCFP approach is better-suited for this application. To improve the error stemming from the semi-infinite approximation, a calibrated correction factor could be applied that varies with proton *z*-coordinate.

Another major limitation of the MCFP approach is the neglect of equilibrium processes for proton interaction with the sensing surface. Whilst this means in theory that all protons would be absorbed given infinite time, in practice, for the MCFP benchmarking simulation without boundary conditions, over 20% of protons had first-passage times of over an hour, with the longest being over 3 years. However, the MCFP simulations will tend to overestimate the signal, particularly after the mixing time of the system and when the number of protons being produced is low, which is when equilibrium effects will come more into play.

**Fig. 4:**
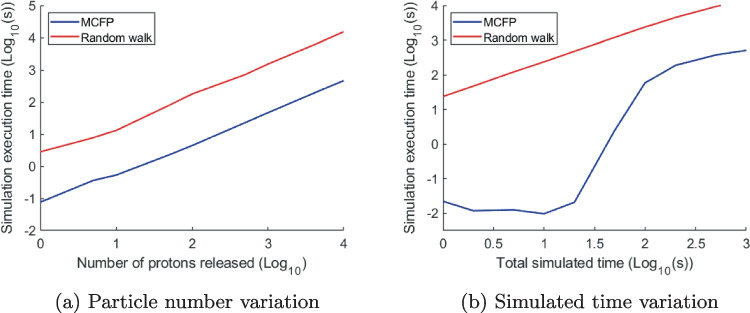
Graphs of simulation execution time for MCFP and random walk algorithms against varying **a**) number of particles released and **b**) total simulated time

#### Simulation runtime

The execution speed of the MCFP algorithm against the benchmark random walk algorithm was tested against a variety of factors, first with the number of particles being simulated (chamber dimension 5mm, total time 1000s). Figure 4a displays the results and demonstrates the quicker runtime of the MCFP simulations, which is consistently over an order of magnitude quicker.

The tests were then repeated with variable chamber dimension (particle number $1\times 10^4$) and the proton release point within the centre of the chamber. The execution time for both simulation approaches was largely invariant with chamber dimension, with the MCFP algorithm again around an order of magnitude quicker than the random walk. This invariance is caused due to the time step of the random walk algorithm and the proportion of protons reflecting off the sidewalls in the MCFP algorithm, both scaling with chamber dimension.

The tests varying the total time that is simulated, contained in Fig. 4b, demonstrate that the MCFP algorithm is significantly faster than the random walk for simulation times less than the mixing time of the system. After the mixing time, it is expected that most protons will interact with the sidewalls and thus a high proportion will have their lateral diffusion determined numerically. But before this point, the majority of the protons have their lateral diffusion calculated with the analytical formulation, which is much quicker. Thus, the execution time for the MCFP algorithm is up to 4 orders of magnitude quicker than the random walk when the total simulated time is 20s. By the end of the longest simulation, the execution time of both algorithms is beginning to saturate, as all released protons are reaching the sensing surface, with the MCFP algorithm around 1.5 orders of magnitude quicker.

To demonstrate a scenario more closely resembling the experiments, the cumulative execution time was tracked for a sequence of ten proton releases every 100s (particle number $1\times 10^4$, total time 1000s). In this case, whilst the MCFP algorithm’s execution time is linear, the random walk algorithm displays a squared relationship, as it does not finish tracking all protons before the next release, meaning that there is an accumulation of particles to track. By the end of the simulation, the MCFP algorithm is an order of magnitude faster than the random walk.

**Fig. 5:**
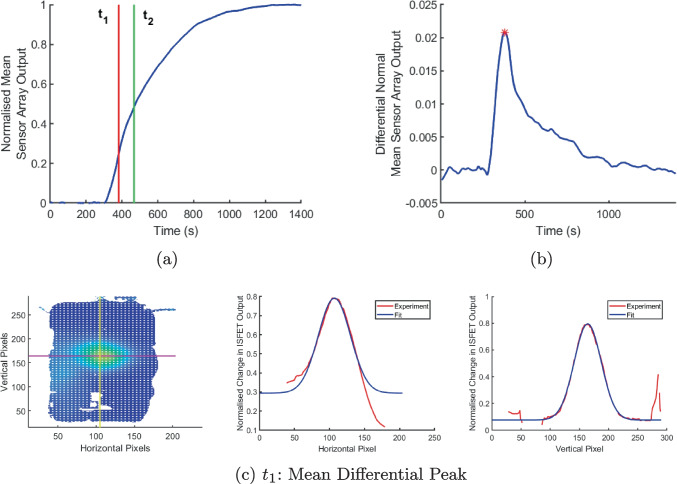
Graphs demonstrating data processing for an experiment with the capillary at 1.2*mm* from the microchip surface. **a**) The drift-corrected mean change in sensor output for all active pixels and **b**) its differential with the peak identified ($t_1$; **red marker**) and the HWHM added to $t_1$ to give $t_2$. The spatial peak within the 2D sensor array is identified at $t_1$ (**c**). For both (purple) horizontal and (yellow) vertical cross-sections centred on the peak, Gaussian fits are made to the sensor output against pixel number, omitting outliers near the periphery of the array

**Fig. 6:**
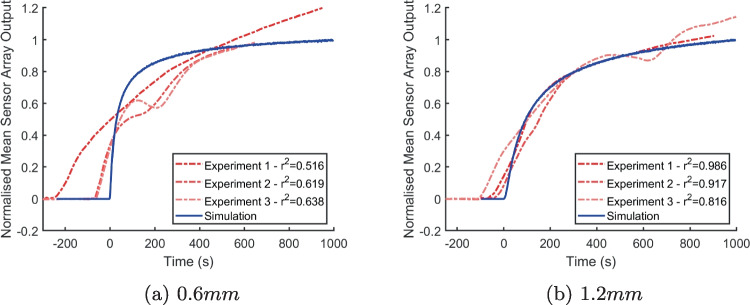
Graphs of normalised sensor array mean output against time with experimental data (red) compared against simulated data (blue) for reflecting sidewalls, with the capillary at **a**) 0.6mm and **b**) 1.2mm

**Fig. 7:**
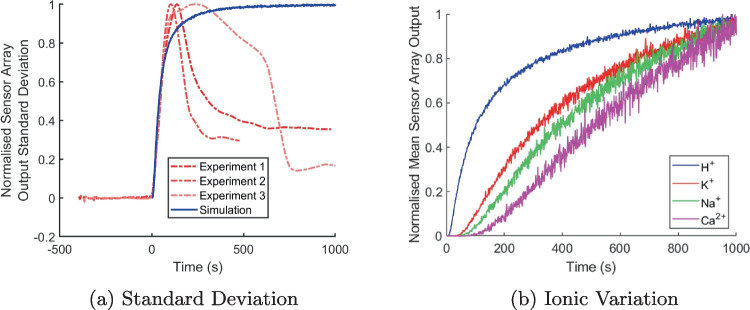
Graphs of **a**) the standard deviation of normalised sensor output against time for the experiments (red) with a capillary height of 1.2mm and the equivalent simulation (blue) and **b**) the variation of normalised sensor array mean output against time with different ionic species

### Experiments

#### Temporal signals

An example of the data processing procedure for experiments is provided in Fig. 5. MCFP simulations showed close agreement with the experiments with the capillary at 1.2mm from the microchip, as presented in Fig. 6, achieving an average $r^2$ value of 0.906±0.070. The experiments with the capillary at a height of 0.6mm did not see the same level of agreement between simulations and experiments, with an average $r^2$ value of 0.591±0.054. This provides an average $r^2$ over all experiments of 0.749±0.170.

The lower agreement of experiments at 0.6mm could be caused by the increased distance that the capillary has to travel within the solution to reach its resting position, inducing greater fluid flow within the chamber. The Reynolds number for these experiments is estimated to be around 1, which suggests laminar fluid flow with stronger viscous effects. Moving mesh COMSOL simulations were performed to estimate the fluid flow induced by the insertion of the capillary. The capillary was assumed to reach its resting position in 1s, giving it a maximum velocity of $4.4\,mms^{-1}$.

The maximum fluid flow was $4.91\,mms^{-1}$ near the tip of the capillary at the moment it comes to rest. This shows that within the first 1s of the experiment, fluid motion is expected to dominate over diffusive motion, which has a mean expected displacement of around $100\,\mu m$ in that time, an order of magnitude lower than the displacement expected from the fluid motion. However, within 1s of movement stopping, the fluid flow is reduced to $7.45\times 10^{-2}\,mms^{-1}$, and therefore, diffusive motion is expected to be significantly more dominant than fluid motion beyond the first recorded frame from the experiments after the capillary is placed in the well.

Though little convection is anticipated, with the entire experimental setup taking place at ambient room temperature, the open top of the well allows evaporative cooling. This was estimated using COMSOL simulations to induce a temperature difference of 1.35 $^{\circ }$C and negligible convective fluid flow rates in the order of $1\times 10^{-8}ms^{-1}$.

Over short times, the simulations accurately represent the standard deviation against time, as illustrated in Fig. 7a. All experiments then display a distinct peak around 100-150s, which coincides with the mixing time of the system. The standard deviation does not return to the baseline level, illustrating that spatial patterns persist on the ISFET array even as the system moves towards equilibrium. This could be caused by protons diffusing within the passivation layer and reacting with buried sites, thereby not taking part in equilibrium processes and forming a new, quasi-trapped charge. This ‘sensor memory’ could be useful for detecting spatial signals.

**Table 4:** A table showing the $\sigma ^2$ parameter of the Gaussian fits of experimental and simulated sensor output in horizontal and vertical directions, centred on the signal peak of the 2D sensor array and taken at the time point for the peak of mean array output differential, and this time point plus the HWHM for the peak of mean array output differential. The uncertainties are provided as the standard deviation of the populations (n=3)

Height (*mm*)		${t_1}$		${t_2}$	
	Direction	Experiment	Simulation	Experiment	Simulation
0.6	Horizontal	35.607 (±3.576)	17.640 (±1.419)	38.679 (±0.996)	22.688 (±1.899)
	Vertical	35.567 (±4.382)	31.060 (±2.766)	46.115 (±8.842)	33.072 (±0.168)
1.2	Horizontal	38.896 (±2.403)	23.771 (±1.139)	51.429 (±3.056)	29.055 (±0.872)
	Vertical	40.795 (±4.806)	38.388 (±0.252)	43.240 (±11.754)	47.752 (±0.615)

On top of the equilibrium processes that drive signal homogenisation across the array, sensor saturation and non-linearity could play a role in the decrease of standard deviation. Currently, these are not taken into account within the model, which would impact results for cases with a starting pH outside 2–10 or for big pH changes. As the difference in well and capillary pH decreases, the only change in the simulation is the decrease in diffusive ion flux emanating from the capillary opening. The simulation has neglected the concentration dependence of diffusivity or any interactions between particles in the electrolyte that may also reduce diffusivity. This is valid in the dilute aqueous solutions used in the experiments. Cases with a well pH that is lower than the pH of the capillary would have to be modelled using the diffusion characteristics of the hydroxide ($OH^-$) ion.

#### Ionic variation

The simulations for the best-performing experiments, with a capillary height of 1.2mm, were repeated for different ionic species—$K^+$ ($D=1.96\times 10^{-9}\,m^2s^{-1}$), $Na^+$ ($D=1.33\times 10^{-9}\,m^2s^{-1}$), and $Ca^{2+}$ ($D=0.79\times 10^{-9}\,m^2s^{-1}$) [31]—that are pertinent to other ISFET sensing applications, particularly neural recording, to demonstrate how the temporal signal is influenced by diffusivity.

The results, which are displayed in Fig. 7b, illustrate the slower accumulation of signal on the sensing array as ion diffusivity decreases, with the signal from $K^+$ lagging $H^+$ by 74s at a value of 10% the final value at 1000s, whilst for $Ca^{2+}$, the lag is 177s. The mean sensing array output at 1000s for $K^+$ and $Ca^{2+}$ is 13.1% and 3.3% that of $H^+$, respectively. These results suggest increased difficulty in detecting changes in the concentration of ions with lower diffusivity because of the more gradual accumulation of signal. Such slow signal changes are hard to discern against the temporal drift experienced by ISFETs.

#### Spatial signals

The values for $\sigma ^2$ of the Gaussian fits to the spatial distributions of signal across the sensing array are collated in Table 4. The simulations, which consider only diffusive transport, underestimate the spread of the signal across the array in all but one case. The vertical cross-sections are in closer agreement than the horizontal, with all four vertical values within two standard deviations of the simulations. This potentially highlights errors stemming from the boundaries, as the vertical dimension is larger than the horizontal and thus protons are less likely to interact with the chamber wall, avoiding the associated simulation errors. The simulations overestimate the difference in $\sigma ^2$ between vertical and horizontal directions. Particularly before the mixing time (100s, $t_1$), the spread of the signal is expected to be nearly isotropic. Alternative strategies for modelling the sidewalls are required, possibly utilising probabilistic absorption, which would provide a more realistic boundary condition than the perfectly reflecting case.

The proportional increase in $\sigma ^2$ values when transitioning from $t_1$ to $t_2$ (at both 0.6mm and 1.2mm capillary height) and from a capillary height of 0.6 to 1.2mm (at both $t_1$ and $t_2$) was consistent between experiments and simulations to within two standard deviations in all eight cases. However, there was no consistency between whether the simulations over- or underestimated the increase, or whether the horizontal or vertical directions saw the largest increase. The standard deviations on the experimental values are large owing to the small number of samples (n=3).

## Conclusion

In this paper, the novel MCFP simulation approach for proton diffusion and ISFET pH sensing was presented, which used random sampling of the first-passage distribution with the semi-infinite approximation. When benchmarked against an equivalent random walk simulation, MCFP simulations were validated in cases without boundaries and with reflecting boundary conditions. Significantly, the MCFP simulations were over an order of magnitude quicker than the random walk, increasing to four orders of magnitude quicker before the mixing time.

MCFP simulations were compared against experimental data from a microchip-based ISFET array with a localised pH change produced using a glass capillary. MCFP simulations demonstrated an ability to recreate the temporal signals, but underestimated the spread of spatial signal across the array.

Overall, the MCFP approach has demonstrated its utility in simulating proton transport and ISFET array pH sensing and will be the optimal simulation choice for high throughput applications.

## Data Availability

Data will be made available upon request.
